# Predictive value of thyroid hormone levels for postpartum hemorrhage in hypothyroidism-complicated pregnancy

**DOI:** 10.3389/fendo.2025.1664539

**Published:** 2026-01-13

**Authors:** Yulu Zhu, Liyang Liu, Cheng Wang, Xia Chen, Chunyan Huang

**Affiliations:** 1 Department of Obstetrics and Gynecology, The Affiliated Hospital of Jiangnan University, Wuxi, Jiangsu, China; 2 The Second Obstetrics Department, The Affiliated Hospital of Jiangnan University, Wuxi, Jiangsu, China

**Keywords:** HDP, hypothyroidism, postpartum hemorrhage, pregnancy, thyroid hormone levels

## Abstract

**Objective:**

To explore the predictive value of thyroid hormone (TH) related indices for postpartum hemorrhage (PPH) in pregnant women with hypothyroidism-complicated pregnancy (HCP).

**Methods:**

273 pregnant women with HCP admitted to our hospital from January 2021 to December 2024 were retrospectively selected. The patients were divided into PPH group (n=50) and non-PPH group (n=223) based on whether PPH occurred. The influencing factors were analyzed with univariate and Binary logistic regression analysis. The receiver operating characteristic (ROC) curve was used to evaluate the predictive value of TH related indices for PPH in pregnant women with HCP.

**Results:**

There were statistically significant differences in manual placental removal, delivery conditions, fetal weight >4000g, TSH, and FT4 (*P* < 0.05). The results of Binary logistic regression analysis showed that delivery conditions, TSH, and FT4 were independent influencing factors for PPH in pregnant women with HCP (*P* < 0.05). The ROC analysis results showed that the area under the curve for TSH+FT4 was 0.823, with a standard error of 0.032 (95% CI: 0.761~0.886), Youden index=0.48, sensitivity of 66.00%, and specificity of 81.61%. Patients with high TSH and low FT4 levels had lower Apgar scores for newborns and higher rates of postpartum hemorrhage.

**Conclusion:**

TSH and FT4 have certain value in predicting PPH in pregnant women with HCP, and it is recommended to include them in predictive indices/models.

## 1 Introduction

Pregnancy is a critical stage in a woman’s life, during which maternal physiology undergoes complex changes to meet the developmental needs of the fetus. During this period, the endocrine system, especially the secretion and regulation of thyroid hormones, is crucial. Thyroid hormones are indispensable for normal pregnancy and fetal development, and the maternal thyroid makes numerous adaptive changes. However, if the thyroid gland fails to adapt to these changes, thyroid disorders may arise, with hypothyroidism being a common occurrence. Studies have shown (1, 2) that overt hypothyroidism during pregnancy poses significant risks. It not only increases the risks of miscarriage, gestational hypertension, gestational diabetes, and various obstetric complications, but is also associated with such severe adverse outcomes as fetal death, significantly increasing the likelihood of PPH.

Postpartum hemorrhage (PPH) is a common and serious complication of childbirth, with incidence rates of 2-4% after vaginal delivery and 6% after cesarean section (3). The causes of PPH are diverse, with uterine atony being the primary factor, accounting for over 50% of cases, followed by retained placenta, genital tract lacerations, coagulation problems, and uterine rupture (4). PPH has serious personal and societal impacts on pregnant women, potentially leading to chronic illness, disability, or even death, while society may face issues like impaired child growth and liver function. Globally, the incidence and mortality rates of PPH are high, making it a leading cause of maternal death during pregnancy (5).

Given the close link between hypothyroidism-complicated pregnancy (HCP) and PPH, coupled with the severe consequences of PPH, identifying effective indicators to predict the risk of PPH in pregnant women with hypothyroidism-complicated pregnancy holds significant clinical importance. Thyroid hormone related index, as a crucial parameter reflecting thyroid function status, may play a vital role in predicting PPH. By delving into the relationship between these indices and PPH, it is hoped that more accurate predictive tools can be provided to clinicians, enabling timely intervention to reduce the risk of PPH occurrence, improve pregnancy outcomes in pregnant women with HCP, and safeguard maternal and infant health. Therefore, this study aims to explore the predictive value of the Thyroid Hormone Levels for PPH in pregnant women with HCP, providing a scientific basis for clinical practices.

## 2 Methods

### 2.1 Research objects

For this study, a retrospective selection was made of 300 pregnant women with HCP admitted to our hospital from January 2021 to December 2024. After screening, 273 patients were included (273>181, meeting the sample size criteria). The patients were divided into PPH group (n=50) and non-PPH group (n=223) based on whether PPH occurred, with the criteria being excessive postpartum bleeding after childbirth (more than 500 milliliters for vaginal delivery or more than 1000 milliliters for cesarean section) (3, 6, 7). The inclusion criteria were as follows: (1) All patients met the clinical diagnosis of hypothyroidism during pregnancy (8, 9). There are symptoms and signs of hypothyroidism. Serum TSH is elevated (normal reference value 0.3–4.8 mIU/L), TT4 [normal value 64–154 nmol/L (5–12 μg/dl)], FT4 is decreased [normal value 9–25 pmol/L (0.7–1.9 ng/dl)].(2) All patients underwent anti-thyroid antibody testing. (3) Patients > 18 years old. (4) Patients had no personal history of thyroid disease. (5) Singleton pregnancies. The exclusion criteria were as follows: (1) Patients with significant organ diseases like heart, lung, or kidney conditions. (2) Patients with mental disorders. (3) Patients on long-term anticoagulant therapy. (4) Patients with incomplete clinical data. (5) Women using medications or suffering from chronic diseases (hypertension, heart disease, diabetes, or autoimmune diseases) that could affect thyroid function.

### 2.2 Research methods

#### 2.2.1 Data collection

Patient age, body mass index (BMI), history of hypertension, and general information were collected through the electronic medical records system. All subjects underwent routine prenatal care to assess thyroid function, including testing for parathyroid hormone (PTH), thyroid-stimulating hormone (TSH), free thyroxine (FT4), free triiodothyronine (FT3) with radioimmunoassay, and calculation of the patient’s thyrotroph thyroxine resistance index (TT4RI) and TSH index (TSHI). All thyroid indicators were collected from the third trimester in the third trimester of pregnancy.

#### 2.2.2 Definitions of terms

Primiparous women are those who have never given birth before (parity of zero). Multiparous women are those who have given birth once before (parity of one). Grand multiparous women are those who have given birth two or more times (parity of two or more) (10).

### 2.3 Statistical analysis

The experimental data collected were analyzed with SPSS 27.0 (International Business Machines Corporation, Armonk, New York, USA). The Shapiro-Wilk test was used for normality testing. For normally distributed continuous data, results were presented as $\bar{\text{X}}\pm S$, and comparisons were made with independent samples t-test. For non-normally distributed data, results were expressed as the median with interquartile range (IQR), denoted as MQ2 (Q1, Q3), and analyzed with the Mann-Whitney U test. Categorical data were presented as frequencies or rates, and comparisons were conducted with χ^2^ test or Fisher’s exact test. Influencing factors were analyzed with univariate and Binary logistic regression analysis. The receiver operating characteristic (ROC) curve was employed to evaluate the predictive value of theThyroid Hormone Levels for PPH in pregnant women with HCP. A significance level of *P* < 0.05 was considered statistically significant for differences.

## 3 Results

### 3.1 Univariate analysis of factors influencing PPH in pregnant women with HCP

Statistical significance was observed in the comparison of manual placental removal, delivery conditions, fetal weight >4000g, TSH, and FT4 (*P* < 0.05), as shown in **Table 1**.

**Table 1:** Univariate analysis of factors influencing PPH in pregnant women with HCP.

Indicator	Classification	PPH group (n=50)	Non-PPH group (n=223)	*Z/t/χ*^2^ value	*P* value
Age (years)		31.54 ± 3.51	31.66 ± 3.57	0.216	0.830
Pre-pregnancy BMI (kg/m^2^)		21.54 ± 1.35	21.79 ± 1.64	1.004	0.316
Manual Placental Removal	Yes	7	10	6.333	0.012
	No	43	213		
Delivery Conditions	Primiparous	25	72	5.594	0.018
	Non-Primiparous	25	151		
SBP (mmHg)		112.56 ± 10.36	112.19± 11.49	0.209	0.834
DBP (mmHg)		70.69 ± 8.98	70.45 ± 8.74	0.175	0.862
Fetal weight >4000g	Yes	6	9	4.989	0.026
	No	44	214		
PTH (pg/L)		30.67 ± 5.64	30.43 ± 5.77	0.267	0.790
TSH (mIU/L)		4.01(3.38, 4.68)	3.31(2.92, 3.77)	-5.582	<0.001
FT4 (ng/dL)		1.19(0.90,1.40)	1.52(1.18, 1.93)	-5.140	<0.001
FT3 (pmol/L)		5.52 ± 1.16	5.77 ± 1.17	1.368	0.173
TT4RI		36.01 ± 9.24	36.98 ± 9.11	0.679	0.498
TSHI		2.91 ± 0.43	2.97 ± 0.52	0.759	0.448

Body Mass Index (BMI); Thyrotroph Thyroxine Resistance Index (TT4RI), Thyroid-stimulating hormone Index (TSHI), Thyroid Feedback Quantile-based Index (TFQI), which were calculated based on serum free triiodothyronine (FT3), free thyroxine (FT4), and thyroid-stimulating hormone (TSH); Anti-thyroperoxidase autoantibody (TPO-Ab); Parathyroid hormone (PTH); Systolic Blood Pressure (SBP); Diastolic Blood Pressure (DBP).

### 3.2 Binary logistic regression analysis of factors influencing PPH in pregnant women with HCP

Using the significant variables identified in the univariate analysis as independent variables, a collinearity check was conducted after assigning values to them. The occurrence of PPH was considered as the dependent variable (PPH = 1, non-PPH=0) for analysis. The results of the Binary logistic regression analysis indicated that delivery conditions, TSH, and FT4 were independent influencing factors for PPH in pregnant women with HCP (*P* < 0.05), as shown in **Table 2**, **Table 3**.

**Table 2:** Variable assignment.

Influencing factor	Assignment
Manual Placental Removal	No=0, Yes=1
Delivery Conditions	Non-primiparous=0, Primiparous=1
Fetal weight >4000g	No=0, Yes=1
TSH	Original Value
FT4	Original Value

**Table 3:** Binary logistic regression analysis of factors influencing PPH in pregnant women with HCP.

Factor	β	Standard error	Wald	P	Exp(β)	95%CI		Collinearity	
						Lower limit	Upper limit	Tolerance	VIF
Manual Placental Removal	1.106	0.596	3.436	0.064	3.021	0.939	9.723	0.985	1.015
Delivery Condition	0.832	0.368	5.117	0.024	2.297	1.117	4.722	0.987	1.013
Fetal weight >4000g	1.227	0.654	3.519	0.061	3.410	0.946	12.283	0.987	1.013
TSH	1.442	0.294	24.068	0.000	4.229	2.377	7.523	0.990	1.010
FT4	-1.571	0.487	10.405	<0.001	0.208	0.080	0.540	0.983	1.017
Constant	-4.972	1.267	15.407	<0.001	0.007	–	–	–	–

### 3.3 ROC curve analysis of predictive value of indicators

The ROC analysis results indicated that the predicted area under the curve for TSH was 0.753, with a standard error of 0.042 (95% CI: 0.669-0.836), Youden index of 0.44. At that point, the sensitivity was 58.00%, and the specificity was 71.75%. For FT4, the predicted area under the curve was 0.733, with a standard error of 0.035 (95% CI: 0.664-0.801), Youden index of 0.37. In this case, the sensitivity was 94.00%, and the specificity was 42.60%.

**Figure 1:**
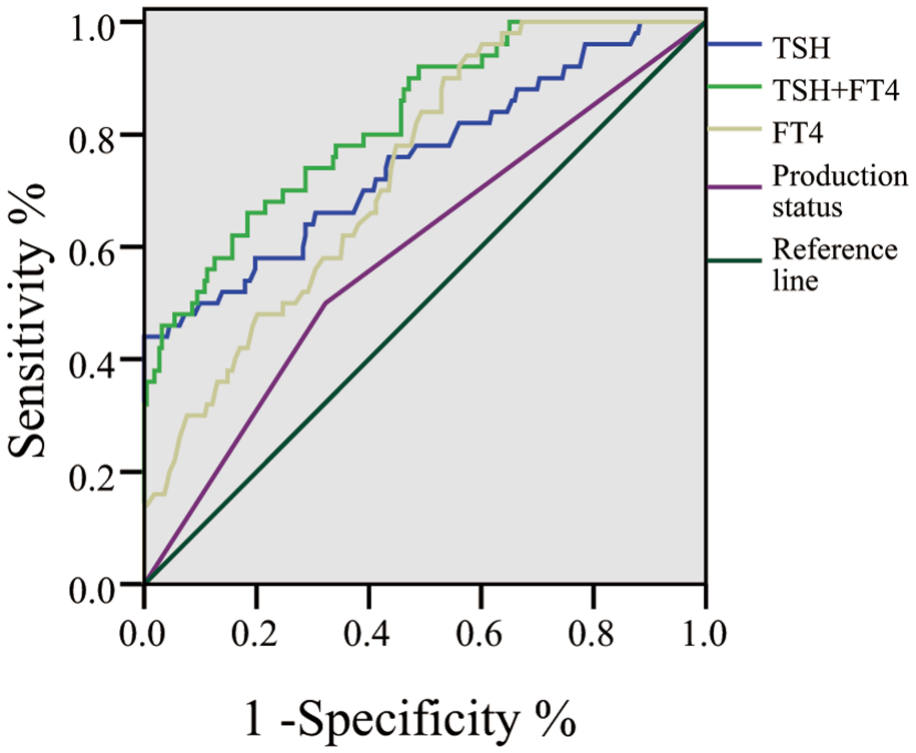
ROC Curve.

The combined predicted value of TSH+ FT4 was tested using logistic regression probability. The combined TSH+FT4 predicted area under the curve was 0.823, with a standard error of 0.032 (95% CI: 0.761-0.886), Youden index of 0.48. At that moment, the sensitivity was 66.00%, and the specificity was 81.61%. Based on the AUC results, it can be observed that the joint predictive value of TSH+ FT4 is the highest, Based on the value at the best threshold Youden Index on the ROC curve as the best cutoff value, the best cutoff values for TSH and FT4 are 4.22 (mIU/L) and 1.62(ng/dL) respectively. As shown in **Table 4**; **Figure 1** for details.

**Table 4:** ROC analysis results.

Indicator	AUC	Standard error	95%CI	Youden	Sensitivity	Specificity	Cut-off value	*P* value
Delivery Condition	0.589	0.045	0.500~0.678	0.18	50.00	67.71	–	0.050
TSH	0.753	0.042	0.669~0.836	0.44	58.00	71.75	4.22	<0.001
FT4	0.733	0.035	0.664~0.801	0.37	94.00	42.60	1.62	<0.001
TSH+FT4	0.823	0.032	0.761~0.886	0.48	66.00	81.61	–	<0.001

### 3.4 Comparison of pregnancy outcomes in patients with different TSH and FT4 levels

Patients were classified based on the optimal cutoff values of TSH and FT4. Patients with TSH ≤ 4.22 were categorized as low TSH, while those with TSH > 4.22 were classified as high TSH. Patients with FT4 ≤ 1.62 were considered as low FT4, and those with FT4 > 1.62 were classified as high FT4. Patients were divided into four groups based on these two indicators. There were statistically significant differences (*P* < 0.05) in the comparison of low Apgar scores and postpartum hemorrhage among the groups. The group with high TSH and low FT4 showed higher rates of low Apgar scores in newborns and postpartum hemorrhage. See **Table 5** for details.

**Table 5:** Comparison of pregnancy outcomes in patients with different TSH and FT4 levels.

Indicator	Classification	Low TSH, low FT4 (n=154)	High TSH, low FT4 (n=21)	Low TSH, high FT4 (n=101)	High TSH, high FT4 (n=1)	*t/χ*^2^ value	*P* value
Preterm Birth (Gestational Age <37 Weeks)	Yes	36	8	24	0	2.554	0.466
	No	118	13	77	1		
Low Apgar Score	Yes	11	3	7	0	13.745	0.003
	No	143	18	14	1		
Postpartum Hemorrhage	Yes	26	21	3	1	113.76	<0.001
	No	128	0	98	0		

## 4 Discussion

This study conducted a retrospective analysis of data from pregnant women with HCP treated at our hospital during a specific period. Thyroid dysfunction may indirectly participate in the pathogenesis of PPH by affecting vascular endothelial function, coagulation system and autonomic nervous balance. Specifically, thyroid hormone can regulate the activity of vascular endothelial nitric oxide synthase and promote vasodilation. Its abnormality may lead to an imbalance between vasoconstriction/anti-proliferation and relaxation/pro-proliferation, triggering pulmonary vascular remodeling; at the same time, hyperthyroidism is associated with hypercoagulability, while hypothyroidism may increase the risk of thrombosis due to abnormal coagulation function, and pulmonary vascular lesions and thrombosis are both core mechanisms of PPH. In addition, the regulation of autonomic nerves by thyroid hormones may indirectly affect pulmonary vascular tone and further participate in the pathophysiological process of PPH. It delved into the predictive value of Thyroid Hormone Levels for PPH and found that TSH and FT4 have certain predictive value.

Previously, a study by Vamja R et al. (11) indicated that women with hypothyroidism have a higher risk of postpartum hemorrhage compared to those with normal thyroid function. However, there has been no further exploration related to Thyroid Hormone Levels. In this study, in univariate analysis, variables including manual placental removal, delivery conditions, fetal weight > 4000g, TSH, and FT4 showed statistically significant differences between the PPH group and the non-PPH group. Manual placental removal may be related to impaired uterine contraction function. During placental removal, mechanical stimulation of the uterine wall may lead to impaired uterine contraction coordination, thereby increasing the risk of postpartum hemorrhage (12). Delivery conditions encompass various aspects such as labor progression and mode of delivery. Complex delivery conditions may subject pregnant women to greater stress, affecting physiological functions including coagulation mechanisms and uterine contraction, thus being associated with the occurrence of postpartum hemorrhage. Fetal weight > 4000g indicates macrosomia, which during delivery may lead to birth canal injuries, excessive uterine expansion, affecting uterine contraction recovery, and increasing the probability of postpartum hemorrhage (13). TSH and FT4, as Thyroid Hormone Levels, their levels changing in association with postpartum hemorrhage, indicate that thyroid function status may have a significant impact on the occurrence of postpartum hemorrhage.

Binary logistic regression analysis further clarified that delivery conditions, TSH, and FT4 are independent influencing factors for the occurrence of PPH in pregnant women with HCP. Previous studies by Dinlen Fettah N et al. (14) have shown a higher incidence of postpartum hemorrhage in patients with subclinical hypothyroidism (SCH) with abnormal FT4 and TSH levels, which aligns closely with the current results. From a physiological perspective, abnormal levels of TSH and FT4 may impact the occurrence of postpartum hemorrhage through various pathways. Thyroid hormones play a crucial role in maintaining normal metabolism, cardiovascular function, and hemostasis balance (15). Elevated TSH and decreased FT4 levels indicate hypothyroid status. Hypothyroidism can slow down metabolism, affecting the energy metabolism and contractile function of uterine smooth muscle cells, leading to weak uterine contractions and thereby increasing the risk of postpartum hemorrhage. Previously, research by Veltri F et al. (16) indicated a negative correlation between postpartum hemorrhage and serum FT4 levels (OR: 0.35; 95% CI: 0.13-0.96), which is consistent with the current study where patients in the PPH group had lower FT4 values. Additionally, thyroid hormones may influence the production of clotting factors. In hypothyroidism, alterations in the synthesis and activity of clotting factors may occur, leading to coagulation disorders, which is also a potential factor contributing to the occurrence of postpartum hemorrhage ( (17).

The ROC analysis results indicate that the combined prediction of TSH + FT4 has an area under the curve of 0.823, suggesting that this combined indicator has a certain predictive capability for PPH in pregnant women with HCP. With a sensitivity of 66.00% and specificity of 81.61%, this combined indicator demonstrates a certain level of accuracy in identifying pregnant women at risk of developing PPH, being able to distinguish to some extent between high-risk and low-risk populations. Furthermore, the study also found that newborns of patients in the high TSH, low FT4 group had lower Apgar scores and a higher incidence of postpartum hemorrhage. The Apgar score of a newborn is an essential indicator for assessing the health status of a newborn after birth. The low Apgar scores in the high TSH, low FT4 group suggest that hypothyroidism may not only affect the coagulation and uterine contraction function of pregnant women themselves but also have adverse effects on the intrauterine environment of the fetus, thus impacting the health status of the newborn. Prior research by Li P et al. (18) has shown a higher incidence of postpartum hemorrhage in cases of maternal iodine deficiency (IMH), which aligns closely with the findings of this study.

Overall, thyroid hormones play a wide-ranging and crucial role in human physiology, and there are various potential biological mechanisms linking them to PPH. From the perspective of vascular function, thyroid hormones can regulate endothelial nitric oxide synthase activity and promote vasodilation. When thyroid function is abnormal, this regulation becomes imbalanced, disrupting the balance between vasoconstriction/anti-proliferation and vasodilation/proliferation, leading to pulmonary vascular remodeling, affecting placental blood flow perfusion, and increasing the risk of hemorrhage. Regarding the coagulation system, hyperthyroidism places the body in a hypercoagulable state, while hypothyroidism may increase the risk of thrombosis due to coagulation abnormalities. Pulmonary vascular lesions and thrombosis are both core mechanisms of PPH, and thyroid hormones indirectly contribute to the occurrence of PPH by affecting the coagulation system. The autonomic nervous system is also regulated by thyroid hormones, and its abnormalities may affect pulmonary vascular tone, further participating in the pathophysiological process of PPH. In addition, thyroid hormones significantly influence the energy metabolism and contractile function of uterine smooth muscle cells. Hypothyroidism reduces uterine contractility, which is unfavorable for hemostasis after placental detachment. However, this study is a retrospective study, mainly based on existing clinical data. During data collection, it is difficult to comprehensively and accurately obtain detailed information related to the aforementioned biological mechanisms, such as specific indicators of endothelial function, precise levels of coagulation factors, and the specific regulatory state of the autonomic nervous system. Moreover, retrospective studies cannot dynamically monitor or intervene in the subjects’ thyroid hormone levels, making it difficult to determine the causal relationship between changes in thyroid hormone levels and PPH, and it is also impossible to rule out other potential confounding factors, thereby preventing the identification of specific biological mechanisms. Future studies should select pregnant women from multiple medical centers as subjects, stratify them according to thyroid hormone levels, and establish a prospective cohort. Thyroid hormone levels (TSH, FT4, etc.) should be regularly monitored during pregnancy, while recording other factors that may influence PPH, such as delivery mode and fetal weight. After delivery, PPH occurrence should be observed, and relevant biological indicators (such as endothelial function markers and coagulation factor levels) should be collected, analyzing the relationship between thyroid hormone levels, these indicators, and PPH, in order to verify the potential biological mechanisms.

However, this study also has certain limitations. Firstly, being a retrospective study, there may be information bias and selection bias, such as the completeness of medical records which could affect the accurate assessment of relevant factors. Secondly, the sample size is relatively limited, which may impact the stability and generalizability of the study results. Future researches could expand the sample size, utilize prospective study designs to more accurately evaluate the predictive value of thyroid hormone related indicators for postpartum hemorrhage. Moreover, this study has limitations in the analysis of risk factors for postpartum hemorrhage. The univariate analysis method is relatively simple and fails to fully include multi-dimensional factors that are strongly related to postpartum hemorrhage, which may lead to insufficient in-depth and accurate exploration of the influence degree of risk factors. Additionally, further exploration of the specific molecular mechanisms through which hypothyroidism influences postpartum hemorrhage could provide a more precise theoretical basis for clinical interventions.

This study also has certain limitations. First, as a retrospective study, there may be selection bias, and the included pregnant women may be limited in terms of region, ethnicity, and economic status, resulting in insufficient sample representativeness. For example, the cesarean section rate in this study was about 30%. A higher proportion of cesarean deliveries may affect the incidence of PPH, leading the results to reflect the characteristics of the cesarean population more than those of the vaginal delivery population, raising questions about applicability and potentially affecting the generalizability of the results. There is also measurement bias, as errors may exist in thyroid hormone level testing, with accuracy potentially varying between different testing methods and reagents, leading to less precise measurements. It is estimated that this measurement bias could result in about a 10% deviation in estimating the relationship between thyroid hormone levels and PPH. Additionally, some potential confounding factors that might simultaneously affect thyroid hormone levels and PPH occurrence were not adequately considered. If not properly controlled, this could either overestimate or underestimate the relationship between thyroid hormone levels and PPH, limiting a thorough and accurate exploration of the impact of risk factors. A single-center study may offer consistency in diagnostic and treatment procedures and testing equipment, but lacks multi-center data validation, which may fail to reflect the actual situation across different medical institutions and affect the universality and reliability of the results. Furthermore, the study’s method for analyzing risk factors is relatively simple, not fully incorporating multi-dimensional factors, which impacts an in-depth and accurate exploration of the influence of risk factors. Second, the relatively limited sample size may affect the stability and generalizability of the study results. Future research could expand the sample size and use a prospective study design to more accurately assess the predictive value of thyroid hormone-related indicators for postpartum hemorrhage.

## 5 Conclusion

In conclusion, this study highlights the predictive value of TSH and FT4 in assessing the risk of postpartum hemorrhage (PPH) in pregnant women with hypothyroidism-complicated pregnancy (HCP). These findings suggest that TSH and FT4 could be valuable components in predictive models or indicators for PPH. However, given the limitations of our study, further high-quality research is necessary to validate and refine these conclusions. Future studies should aim to strengthen these findings, which could ultimately inform clinical practices, reduce the risk of PPH in women with HCP, and improve both maternal and neonatal outcomes.

## Data Availability

The original contributions presented in the study are included in the article/supplementary material. Further inquiries can be directed to the corresponding author.
